# Cellular Adhesion Molecules in Healthy Subjects: Short Term Variations and Relations to Flow Mediated Dilation

**Published:** 2008-02-14

**Authors:** Ole Eschen, Jeppe Hagstrup Christensen, Claus Dethlefsen, Erik Berg Schmidt

**Affiliations:** 1 Department of Cardiology; 2 Center for Cardiovascular Research and; 3 Department of Nephrology, Aalborg Hospital, Aarhus University Hospital, Aalborg, Denmark

**Keywords:** variation, healthy subjects, cellular adhesion molecules, flow mediated dilation

## Abstract

The objective was primarily to describe short term intra-individual variation in serum levels of soluble adhesion molecules (sCAMs: E-selectin, P-selectin, intercellular adhesion molecule-1(sICAM-1) and vascular cellular adhesion molecule-1(sVCAM-1)) in healthy subjects. Secondly, sCAMs were correlated to brachial artery flow mediated vasodilation (FMD).

Forty healthy subjects aged 24–66 years had sCAMs measured twice with 4 week intervals and short-term intra-individual variation was estimated as variation in the paired measurements after correcting for the analytical precision of the used method. At baseline, brachial FMD was measured.

No difference was observed in mean sCAMs in the whole study group. Estimated intra-subject variations in sCAMs were 7.6–11.3%. In a regression analysis, significant negative association was found between sE-selectin and FMD after controlling for possible confounders (p < 0.04) while no significant correlation could be demonstrated between the other sCAMs and FMD.

In conclusion, short term intra-individual variations in sCAMs were 7.6–11.3% in healthy subjects. We also found a significant negative association between sE-selectin and FMD, indicating an possible association between inflammation and dysfunction of the vascular endothelium; however further studies are required to confirm this preliminary finding.

## Introduction

The recruitment, adhesion and subsequent transendothelial migration of circulating leucocytes is important in the initiation and progression of atherosclerosis (Ross, 1999). These processes are mediated by cellular adhesion molecules expressed by the vascular endothelium. Among these are vascular cell adhesion molecule-1 (VCAM-1), intercellular cell adhesion molecule-1 (ICAM-1), E- and P-selectins. The biological active molecules are trans-membrane proteins, but soluble forms of adhesion molecules (sCAMs) can be measured in plasma. sCAMs are elevated in subjects with traditional risk factors for developing cardiovascular disease (CVD), i.e. hypertension, diabetes, smoking, obesity and dyslipidemia (Celermajer et al. 1993; Chae et al. 2001; Clausen et al. 2000; Straczkowski et al. 2002; Winkelmann et al. 2001). Furthermore, sCAMs are also elevated in autoimmune diseases and in malignant diseases (Dedrick, Bodary and Garovoy, 2003; Kobayashi, Boelte and Lin, 2007; Witkowska and Borawska, 2004).

The biological variability of sCAMs needs to be known and be low enough to enable reliable risk stratification. The intra-individual variability in serum levels of sCAMs has not previously to our knowledge been investigated in detail.

Flow mediated vasodilation (FMD) in the brachial artery is a non-invasive method to access endothelial function. FMD correlate negatively with known risk factors of CVD, such as dyslipidemia, diabetes, hypertension, smoking (Celermajer, Sorensen, Georgakopoulos, Bull, Thomas, Robinson and Deanfield, 1993), adiposity (Brook et al. 2001), and a familial history of CVD (Celermajer et al. 1994). Furthermore, FMD may have prognostic information about future CVD events (Gokce et al. 2003).

In the present study, we have investigated the short time variation in sCAMs in healthy volunteers and (as a pilot study) correlated FMD to plasma levels of sCAMs.

## Methods

### Study population

Forty healthy subjects were recruited from the medical staff, bank employees and students from Aalborg, Denmark. None of the volunteers took any medication or had any known disease. The procedures followed were in accordance with the ethical standards of the responsible Regional Committee on Human Experiments and in accordance with the Helsinki Declaration of 1975 as revised in 1983. All subjects gave written informed consent.

### Measurement of adhesion molecules

Blood samples were taken from the left cubital vein twice with an interval of 4 weeks (and in all women at the same time of menstrual cycle). Venous blood was collected between 08:00 h and 09:30 h after fasting overnight for at least 10 hours. Blood samples were allowed to clot for 1 hour at room temperature, and serum collected after centrifugation at 2500 × g for 20 minutes and stored at −80 °C until analysis. Determination of serum levels of VCAM-1, ICAM-1, E-selectin and P-selectin were performed using commercially available test-kits (From R&D Systems Europe, Ltd.: Catalogue Numbers: DVC00 (sVCAM-1), BBE 1B (sICAM-1), BBE 6(sP-selectin), BBE 2B (sE-selectin)). Analysis was performed as batch-operation using the same analysis-kit. The samples were analyzed twice and the average of the two measurements is reported.

### Flow mediated dilation

Subjects were fasting overnight and instructed to abstain from tobacco smoking and coffee. After blood samples had been drawn from the left arm, subjects were placed supine at rest for 15 minutes in a quiet room at normal room temperature. A continuous ECG was recorded for timing of measurements. The right radial artery was imaged in a longitudinal section 3 cm below the antecubital fossa. The probe was held in a constant position by a stereotactic clamp. Baseline dimensions were recorded at the center of the vessel with optimal contrast between the anterior and posterior vessel wall and the lumen. Doppler blood flow images were recorded from the center of the vessel. Then an arterial occlusion cuff, placed above the antecubital fossa was inflated to 300 mmHg for five minutes. Immediately after deflation of the cuff, a continuous 90 seconds recording of blood flow and vessel dimensions was obtained. The peak diameter of the artery was recorded 60 seconds after deflation of the cuff, and the percentage change from the baseline diameter was calculated.

### Statistical analysis

Analyses were conducted using STATA statistical software, version 9.1. Short term variation in sCAMs was estimated by a paired t-test and coefficient of variance was calculated as standard deviation of differences between paired measurements divided mean difference in sCAMs within-subjects (in sCAMs between baseline and after 4 weeks). Analytical precision was calculated as a coefficient of variation based on double measurements of all blood samples taken at baseline and after 4 weeks (80 double measurements).

Pearson’s correlation coefficient was used as a measure of linear association. Linear regression analysis was conducted to correct for possible confounding effects. Age was included as a numerical variable and age-groups as an indicator variable, allowing for non-linear relation between age and sCAMs. Body Mass Index, serum total cholesterol, systolic blood pressure were included as numerical variables and (current) smoking status and gender as indicator variables. A p-value <0.05 (two tailed) was considered statistically significant.

## Results

Characteristics of the forty participants are given in Table 1. No significant correlation was found between either sCAMs or FMD and blood pressure, BMI, age and serum lipids and lipoproteins. Significant correlation was found between sP-selectin and sE-selectin (r = 0.82, p < 0.001).

### Short term variations in adhesion molecules

The results of measurements of sCAMs with four weeks interval are given in Table 2, and the calculated CV_study_% of the measurements is given. Also the table provides an estimate of the intra-individual variation, CV_intra-subject_% (the best estimate of intra-individual short-time variation), based on the assumption that the measured CV_study_% consists of two components: variation (analytical precision) of the assay (CV_analytical_) and variation in the difference between the paired measurements (intra-individual, CV_intra-subject_). Then intra-individual variation were calculated using CV_study_^2^ = CV_analytical_^2^ + CV_intra-subject_^2^. Overall, the CV_intra-subject_% for each sCAM was between 7.6–11.3%.

### Cellular adhesions molecules and flow mediated dilatation

**Figure 1:**
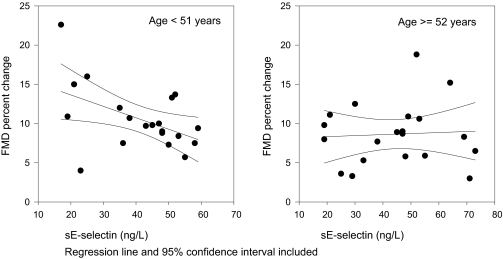
Scatterplot of sE-selection and Flow Mediated Vasodilation (FMD) in the study population dichotomized according to median age.

No significant correlations were found between FMD and each of sCAM, systolic blood pressure, BMI, smoking habits, lipids and lipoproteins or age. By gender, scatter plots of the data revealed that the variability were higher in the females (data not shown). Variation also increased with age. Therefore, a regression analysis was performed with FMD as independent variable and sCAMs, gender and age. Age was included both as a numerical variable and with two age groups according to median age (51 years) as an indicator variable, allowing for non-linear relation between age and sCAMs. This effect remained significant after including following dependent variables (possible confounders): serum total cholesterol, BMI, systolic blood pressure and smoking habits. The results of the regression analysis are given in Table 3. A scatter plot of sE-selectin and FMD divided according to median age is given in Figure 1.

## Discussion

sCAMs may become a routinely used risk marker of preclinical atherosclerosis and therefore information about the within-subject biological variation is essential. In the present study, our primary objective was to estimate within-subject short term biological variation in serum level of sCAMs in healthy volunteers. The estimated within-subject variation coefficients were between 7.6–11.3% for all measured sCAMs. The difference in the study population mean serum sCAMs was negligible (less than 1%) in samples taken 4 weeks apart. Also analytical precision of commercially available analysis assays is excellent (CV% of approximately 2%). We have no knowledge of other studies investigating the short term variation in sCAMs under standardized conditions. However, variations in sCAMs have been investigated both as diurnal variations and variation due to physical or psychological stress. The diurnal intra-subject variation in sCAMs in healthy subjects and in patients with CHD have been reported for P-selectin, ICAM-1 and E-selectin (Osmancik et al. 2004); only P-selectin showed significant diurnal variation (highest in the evening). Effects of factors such a physical or psychological stress may also induce within-subject variations in serum levels of sCAMs of approximately 10% (Dugue, Leppanen and Grasbeck, 1999). Thus, the result may have implications for studies evaluating sCAMs as a marker of risk. An important parameter to consider in the design of clinical trials assessing systemic low grade inflammation in atherosclerosis is the degree of within-subject biological variation. One way to reducing the inherent variability of the technique might be repeated measurements of sCAMs due to the effect of reducing the SD and thereby the sample size estimates. Indeed, our results indicate that repeated measurements of sCAMs may be preferable as the short term intra-individual variation is approximately 10 percent.

In the second part of our study we aimed to investigate an association between two important aspects in the progression of atherosclerosis, that is the lowgrade endothelial/systemic inflammation and the endothelial dysfunction. There is little knowledge of interrelations between FMD and sCAMs in healthy individual. In contrast, several reports have related either FMD or sCAMs to the presence of traditional risk factors for CVD, such as dyslipidemia, diabetes (Clausen, Jacobsen, Rossing, Jensen, Parving and Feldt-Rasmussen, 2000; Ravikumar et al. 2002), hypertension (Chae, Lee, Rifai and Ridker, 2001; Park, Charbonneau and Schiffrin, 2001), smoking (Celermajer, Sorensen, Georgakopoulos, Bull, Thomas, Robinson and Deanfield, 1993; Winkelmann, Boehm, Nauck, Kleist, Marz, Verho, Ranjith and Kneissl, 2001), and adipositas (Brook, Bard, Rubenfire, Ridker and Rajagopalan, 2001; Straczkowski, Lewczuk, Dzienis-Straczkowska, Kowalska, Stepien and Kinalska, 2002). In our study, sE-selectin correlated to FMD. In contrast, Witte (Witte et al. 2003) reported that sICAM-1 and FMD in the brachial artery correlated in 166 healthy individuals, and they also reported that both sICAM-1 and FMD related to the estimated risk of CVD. They did not measure sE-selectin, sP-selectin or sVCAM-1. The reasons for this discrepancy is not clear, but in the study but Witte et al. more subject were included. (In our study, the association between sICAM-1 and FMD came close to significance with a p-value = 0.11). E-selectin is exclusively expressed on endothelial cells (Roldan et al. 2003), in contrast to sP-selectin, sICAM-1 and sVCAM-1. Perhaps sE-selectin is a more specific marker of inflammation in vascular endothelium, because both ICAM-1 and VCAM-1 are expressed on several other cells (e.g. inflammatory leucocytes, smooth muscle cells) and therefore they might be more general markers of universal low grade systemic inflammation (Blankenberg, Barbaux and Tiret, 2003). The major source of sP-selectin is believed to be activated platelets (Blann, Nadar and Lip, 2003), though sP-selectin is also expressed by the activated endothelium. We found a close significant correlation between sP-selectin and sE-selectin. However, after controlling for possible confounders sP-selectin did not correlate to FMD. In early atherosclerosis, also endothelial P-selectin expression is crucial in the progression of the atherosclerotic lesion(Burger and Wagner, 2003), and therefore this close link between endothelial activation (and E-selectin expression) and sP-selectin was expected. The interrelations between sCAMs and endothelial vasodilatory function in healthy subject have been investigated by strain-gauge venous occlusion plethysmography. Holmlund (Holmlund et al. 2002) reported that only sICAM-1 but not sE-selectin or sVCAM-1 correlated to endothelial vasodilatory function in 59 healthy subjects. This contrasts our findings. However, both studies indicate that endothelial vasodilatory function and markers of endothelial inflammation are correlated. This also fits the findings that both sCAMs (Blankenberg, Barbaux and Tiret, 2003; Ridker et al. 1998; Ridker, Buring and Rifai, 2001) and FMD (Gokce, Keaney, Jr., Hunter, Watkins, Nedeljkovic, Menzoian and Vita, 2003) may have prognostic information about future CVD.

Limitations of our study are primarily the small number of subjects under investigation. However, the differences in sCAMs between within subject paired measurements showed good approximation to a normal distribution. Population size was of greater importance in the investigation of a possible association between sCAMs and FMD. Because data analysis revealed a marked age-dependency and marked gender dependency on the association between sCAMs and FMD, a regression analysis including possible confounders were performed. However, care should be taken to make firm conclusions based on this part of the study. This part of our study should only be viewed as a pilot study. A large scale study is required to establish an association between sCAMs and FMD.

In conclusion, short term intra-individual coefficients of variation in sCAMs in healthy subjects are estimated to be 7.6–11.3 percent under standardized condition. This intra-subject variation need consideration in evaluation of sCAMs in experimental trials and in clinical use. We also found a weak significant negative correlation between sE-selectin and FMD, indicating an association of dysfunction and inflammation in the vascular endothelium. However, due to the small population size, a larger study is required to establish possible association between FMD and sCAMs.

## Figures and Tables

**Table 1:** Population characteristics at baseline.

Gender (male/female, n)	20/20
Age (years)	48.4 ± 12
BMI (kg/m^2^)	24.0 ± 3
Current smokers (n (percent))	9 (23)
Systolic BP (mmHg)	115 ± 10
Diastolic BP (mmHg)	74 ± 7
Creatinine (μmol/l)	81 ± 13
Fasting glucose (mM)	4.4 ± 0.4
Total cholesterol (mM)	5.2 ± 1.3
Triglycerides (mM)	1.1 ± 0.5
HDL-cholesterol (mM)	1.4 ± 0.3
LDL-cholesterol (mM)	3.3 ± 1.2
sICAM-1 (ng/l)	233 ± 114
sVCAM-1 (ng/l)	515 ± 103
sP-selectin (ng/l)	25 ± 8
sE-selectin (ng/l)	43 ± 15
FMD (percent change)	9.6 ± 4.1

Exact number or mean ± SD.

**Table 2:** Short-time variation in soluble cellular adhesion molecules in 40 healthy subjects (samples taken with 4 weeks interval).

	VCAM-1	P-selectin	E-selectin	ICAM-1
Mean t_Baseline_	515 ± 103	25.0 ± 8.5	42.8 ± 15.4	233 ± 115
Mean t_4 weeks_	515 ± 119	24.5 ± 8.6	42.7 ± 16.5	232 ± 106
Difference ± SD	0.60 ± 49	0.50 ± 2.8	0.10 ± 4.8	0.53 ± 18
CV%	9.6	11.5	11.3	7.9
Analytical CV%†	1.4	2.0	2.1	2.1
Intra-individual CV%‡	9.5	11.3	11.1	7.6

Mean ± SD.

† Based on double measurements of all samples.

‡ Estimated (from: CV_study_^2^ = CV_analytical_ + CV_intra-subject_^2^).

**Table 3:** Regression analysis with Flow Mediated Vasodilation as independent variable and soluble Cellular Adhesion Molecules, gender, age groups, systolic blood pressure, serum total cholesterol, Body Mass Index and smoking habits as dependent variables.

	Flow Mediated Vasodilation (percent change)			
	Coefficient beta	s.d.	t	p-value
sICAM-1†	−0.047	0.02	−1.61	0.12
sVCAM-1†	−0.007	0.01	−0.59	0.56
sP-selectin†	−0.014	0.16	−0.88	0.38
sE-selectin†	−0.16	0.08	−2.06	0.04

† ng/l. Only coefficients for cellular adhesion molecules are given.
